# Symptom recognition and treatment-seeking behaviors in women experiencing acute coronary syndrome for the first time: a qualitative study

**DOI:** 10.1186/s12872-022-02892-3

**Published:** 2022-11-28

**Authors:** Elnaz Asghari, Leila Gholizadeh, Leila Kazami, Mohammadreza Taban Sadeghi, Ahmad Separham, Naser Khezerloy-aghdam

**Affiliations:** 1 Faculty of Nursing and Midwifery, Tabriz University of Medical Science, South Shariati St., Tabriz, 51368 East Azerbaijan Iran; 2 Faculty of Health, University of Technology Sydney, 15 Broadway, Sydney, NSW 2007 Australia; 3 Shahid Madani Hospital, Tabriz University of Medical Science, Tabriz, Iran; 4 Cardiovascular Research Center, Tabriz University of Medical Science, Tabriz, Iran

**Keywords:** Acute coronary syndrome, Qualitative research, Symptom recognition, Prehospital delay, Help-seeking, Women

## Abstract

**Background:**

Women are more likely to delay medical help-seeking for ACS symptoms. Understanding patients’ experience of the symptoms and their response is essential in improving help-seeking behaviors and timely diagnosis and treatment for ACS. This study aimed to explore women’s experience of ACS, their response to the symptoms, and treatment-seeking decisions.

**Methods:**

This qualitative descriptive study was conducted in a tertiary referral specialized heart hospital affiliated with Tabriz University of Medical Sciences, Iran. Participants included 39 women who had experienced ACS for the first time.

**Results:**

Four main themes emerged from the analysis of interview transcripts: (1) the onset of symptoms, (2) the types of symptoms, (3) response to symptoms and (4) arriving at the hospital. These themes and associated sub-themes explained women’s experience of ACS symptoms, their response to the symptoms, and decision to seek medical help.

**Conclusions:**

This study identified and discussed factors contributing to the prehospital delay in women and their decision-making to seek medical care for ACS symptoms. The results are consistent with previous research indicating that ACS symptoms in women are somewhat different from men, and women tend to underestimate their symptoms and attribute them to non-cardiac causes. Women should be supported to develop awareness and understanding of ACS symptoms and appreciate the importance of early treatment-seeking in the disease outcomes.

## Background

Heart disease remains the leading cause of death in men and women worldwide [1]. Acute coronary syndrome (ACS) is an umbrella term used to describe the clinical signs and symptoms associated with impaired blood flow to coronary arteries. It includes unstable angina, non-ST-segment elevation myocardial infarction, and ST-segment elevation myocardial infarction [2, 3]. Chest pain is the most common symptom of ACS among both men and women; however, compared to men, women are more likely to present with pain between the shoulder blades, nausea and vomiting, and shortness of breath, while chest pain and diaphoresis are more common in men [4]. Other symptoms are mainly comparable in prevalence between men and women [4].

ACS is a time-critical condition, which means delays in diagnosis and treatment of the disease can upsurge the risk of heart muscle damage, leading to increased morbidity and mortality [1, 5, 6]. Several patient and service level factors have been identified as affecting the timely diagnosis and treatment of ACS; among them are the patient’s hesitation and delay in seeking medical care is a key factor [7]. Mirzaei et al. found that lack of recognition of ACS symptoms, the discrepancy between actual and expected symptoms, and discrepancy between current symptoms and previous experience of the disease are some factors contributing to delay in timely treatment seeking [6]. Also, a lack of previous experience of ACS affects how the symptoms are interpreted and acted upon by patients [8].

Evidence suggests that women are more likely than men to delay treatment seeking for ACS symptoms. Factors such as anxiety, lack of knowledge of ACS symptoms, and reluctance to bother others contribute to this delay among women [9]. Delay in seeking medical care may partially explain poorer ACS outcomes in female patients compared to their male counterparts [10]. Women also tend to underestimate their risk of cardiovascular disease which can affect their symptom attributions to heart conditions and delay help-seeking [11]. In addition, women are generally underrepresented in cardiovascular research, indicating that clinicians’ knowledge about ACS symptoms, diagnosis, and treatment is predominantly based on research on male populations [12].

However, increasingly sex-related anatomical, physiological, biological, and psychological differences are unfolded, demonstrating differences in disease presentation, diagnosis, management, and prognosis of cardiovascular disease in men and women [3]. For example, it is known that women have smaller coronary lumens and less collateral circulation regardless of body size. These anatomical differences may increase the risk of ischemia in women during periods of physical and mental stress. In addition, the prevalence of psychological diseases, such as depression and anxiety, is significantly higher in women [13]. People with anxiety and depression are more likely to experience angina, shortness of breath, dizziness, nausea, and palpitations, which overlap with ACS, further complicating ACS diagnosis [6, 14]. Also, patients with the comorbidity of depression are more likely to present with severe and recurrent angina [2] and experience poorer outcomes than those without depression [13, 14].

These differences suggest the need for sex and gender-specific approaches to ACS assessment and management [10, 13]. A better understanding of women's experience of ACS, their interpretation of the symptoms, and responses are necessary to educate the public about ACS and the importance of timely treatment. This knowledge can also help paramedics, triage nurses, and clinicians assess, diagnose, and treat this time-sensitive condition promptly [6, 15].

## Methods

### Design

This study aimed to explore women’s experience of first-time ACS and their interpretation of the symptoms and responses. The study adopted a qualitative descriptive design. A qualitative research approach allowed a deeper exploration of participants’ experience of ACS symptoms, including their feelings, perceptions, causal attributions, and responses to the symptoms and treatment-seeking behaviors.

### Ethical consideration

The study obtained ethical approval from the Research Ethics Committee of Tabriz University of Medical Science (IR.TBZMED.REC.1399.911). The researcher provided potential participants with information about the purpose of the study, the data collection method, potential risks and benefits, and the voluntary nature of the study. Informed written consent was obtained from all participants interested in the study, and their privacy and confidentiality were assured. The researchers complied with the national and local guidelines when conducting the interviews during the Covid-19 pandemic.

### Setting

This was conducted in a tertiary heart hospital affiliated with Tabriz University of Medical Sciences, Iran. It is the primary referral public specialized heart hospital in northwest of Iran.

### Sampling

Participants were recruited from the medical wards of the participating hospital. Inclusion criteria were: female patients admitted to the hospital with ACS diagnosis for the first time, aged 18 years and older, those who were able to speak Azerbaijani or Persian languages, and provided consent to the study. All of the eligible patients who were invited to participate in the study accepted our invitation. Exclusion criteria for the study were patients with recurrent ACS, unstable hemodynamics, or mental and cognitive conditions that could have affected their ability to provide informed consent to the research; however, none of the patients screened were excluded.

#### Data collection

Data were collected using semi-structured face-to-face individual interviews. The first author conducted all the interviews between December 2020 and March 2021. Participants determined the time and location of the interviews; they all preferred to complete the interviews in the hospital ward. A semi-structured interview guide was used; interviews began with warm-up questions and then progressed to the main open-ended research questions, for example, "What brought you to the hospital?”, “How did the symptoms start?” and "What did you do when it happened?". Probing questions were asked if needed to elaborate on the details, for example, “how long did it take for you to decide to seek medical help?”. The interviews lasted an average of 48 ± 5.6 min. Researchers were to end the interviews earlier if a participant asked for it or the interviewer felt that the participant was uncomfortable with the interview; however, there was no such case. Researchers ceased participant recruitment after 39 interviews. In the last three interviews, no new codes were obtained. Examination of the themes by the research team also showed no gap. At this stage, the researchers determined that data saturation had been reached; thus, no further interviews were undertaken.

#### Data analysis

All interviews were audio-recorded and transcribed verbatim. The transcriptions were analyzed using the MAXQDA software (2007 version, VERBI Software GmbH, Berlin, Germany). Qualitative analysis of content was used for data analysis [16]. In the first step, data were transcribed into written text. Then, the units of analysis were selected, which included individual themes. In the next step, the units of analysis were classified based on their similarities and differences, leading to the formation of the initial framework of the findings. The constant comparative method was used to classify the semantic units. This method was used both for classification and for merging similar categories. The first two interviews were coded independently by two researchers (EA and RT), and the results were compared and contrasted by the third researcher (LK). Due to a high inter-coder agreement, coding was continued by EA for the remaining interviews, and new concepts and themes were added or merged into the initial framework of the findings. Interview with participant No. 21 was also coded by two researchers (EA and RT), and the results were compared independently by LK. This approach ensured the accuracy, consistency, and integrity of the coding process. Finally, the compatibility of the codes with the study themes and subthemes was re-examined, and the extracted features were compared with each other. The subthemes were compared, and their range was determined. The research team members held several meetings to discuss the pattern and compliance of the codes with the categories and subcategories [16].

## Results

The demographics of participants are presented in Table 1. The mean age of participants was 60 ± 5.2 years, ranging from 51 to 83 years. They were mainly married (71.79%), housewives (64.10%), had health insurance (79.48%), and presented to the emergency department with an accompanying family member (89.74%). A considerable number of participants (30.76%) were illiterate.

**Table 1:** Demographic characteristics of the study participants (n = 39)

	Variable	Frequency	Percentage
Education	Illiterate	12	30.7
	Elementary-mid school	21	53.8
	High school	3	7.6
	University degree	3	7.6
Marital status	Married	28	71.7
	Single	1	2.5
	Widow	10	25.6
Residential status	Rural	19	48.7
	Urban	20	51.2
Job	Self-employed	9	23.0
	Retired	5	12.8
	Housewife	25	64.1
Health insurance	Yes	31	79.4
	No	8	20.5
Arriving at the hospital	Ambulance	22	56.4
	Personal car	17	43.5

The analysis of the interview data revealed four main themes and 11 subthemes. The classifications of the themes and example statements are presented in Table 2.

**Table 2:** Summary of the study themes and sub-themes

Theme	Subtheme	Quotation
The onset of symptoms	Sudden onset of symptoms	*I was eating dinner, suddenly, I felt like the weight of a mountain on my chest. I could see my death. This should tell how it felt (p:31)*
	The gradual development of the symptoms	*I had a headache for a few days; I could not eat or do anything. It was not continuous; no, I just had to rest. It would hurt when I would get up (p: 38)*
The types of	Chest symptoms	*I had the worst world pain in my chest; it was pounding in my left hand. … Squeezing my heart (p: 28)*
	Non-chest symptoms	*I felt suffocated, found it very difficult to breathe, sweated a lot, and my body was weak. I thought I must have gotten the Corona (p: 18)*
	Symptomless	*I was getting prepared for the surgery (knee surgery)… not sure what happened; they referred me here for a heart problem (p: 19)*
Response to symptoms	Using home remedies	*I put a hot brick on my shoulders to help relieve it (pain) (p: 25)*
	Ignoring symptoms	*In older age, you should not pay too much attention to pain; otherwise, you should be in the hospital all the time. You always have pain somewhere (p: 27)*
	Hiding symptoms	*I did not tell anyone about my symptoms. Why should I bother others? Take me to the doctor, buy me medicine, cook for me, I want to rest… (p: 34)*
	Making the decision to seek medical help	*I look after myself. Doctors are here to help us; all these equipment and nurses are here to help us, to take care of us (p: 29)*
		*The pain came, I screamed, my son came to the room, I said I am dying, call the ambulance. It (ambulance) came pretty fast (p: 24)*
Arriving at the hospital	Arriving by ambulance	*It would be dangerous to come (to the hospital) by ourselves. In an ambulance, you are with a health team member, equipment,….(p:37)*
	Using a personal vehicle	*Our house is over that street; walking is even faster than calling an ambulance…like explaining to the operator….. (p: 23)*

### The onset of the ACS symptoms

The onset of ACS symptoms was wide-ranging in terms of the onset time, severity, and type of symptoms. The onset of the symptoms could be day or night time. Only one woman experienced the symptoms while sleeping, and all other women were awake when their symptoms started. The onset of symptoms was sudden in some participants (n = 21) but gradual in others (n = 18). In the sudden onset of symptoms, the severity was high from the start, but in participants with gradual symptoms onset, the severity of the symptoms increased progressively over time.

#### Sudden onset of symptoms

In participants whose symptoms had developed abruptly and acutely, the sudden presentation caused them to focus on symptom relief more than symptom attribution. The following are excerpts from participant interviews.

*I was lying down at night; I felt like the weight of a mountain on my chest. I thought my chest was going to blow up from the pressure anytime (p:17)*

*I was so confused; I didn’t know what was going on. I wasn't sure…should I focus on finding out why I have this pain or just find a solution. Like a cat jumping down from a wall, I was out of breath and shocked (p:7).*

#### Gradual development of the symptoms

Some participants, however, developed the symptoms gradually over time. They experienced symptoms such as shortness of breath, heartburn, or high blood pressure for a couple of days. The gradual and progressive development of the symptoms allowed these women time to analyze and interpret their symptoms. Nevertheless, most women attributed their symptoms to a non-cardiac cause, such as fluctuations in blood glucose level, acid reflux, heavy food, stress, COVID-19, lung problems, muscle overuse, or aging. Acid reflux was the most commonly attributed cause.

*After eating, I had this terrible heartburn, as if a needle was piercing my chest. I said (to my sister): I have a stomach problem; my sister said: no, this is a gallstone problem because it hurts after eating. (P: 15)*

*That day I had cold sweats; my head felt dizzy and heavy; I asked my daughter to check my sugar; I thought my sugar had dropped again. She said my sugar was fine. I thought she had not done it correctly, or perhaps the machine was broken. I said: no, get me some sugar water; I know my sugar has dropped. (P: 35)*

### The types of symptoms

Most women experienced chest symptoms (n = 28), including chest pain, which radiated to the jaw or left arm with or without shortness of breath. However, several women presented with non-chest pain symptoms (n = 8), such as sweating, indigestion, nausea and vomiting, palpitation, and the feeling of numbness. Three participants were symptomless.

#### Experience of chest symptoms

Most women experienced chest symptoms, such as chest pain or chest discomfort with or without shortness of breath. Chest pain was radiating to the jaw or left arm. Participants described their chest symptoms as ‘*feeling a pressure and heaviness on the chest, or a crushing pain*.’ Some participants described their chest symptoms as a feeling of tightness or pressure in the chest. The most common phrases that women used to describe their chest symptoms included: ‘*like a rock on the chest*,' *'felt like a mountain*’, ‘*a very heavy object on the chest*,' *'squeezing the heart tightly in the fist*', and "*as if someone was pressing my heart with his fist*.” Almost all participants who had experienced chest symptoms illustrated their feeling by fisting their hand or pressing their chest with a fist hand and described its severity as ‘*very severe*’ or ‘*deadly*.' Some participants described their chest symptoms as ‘*sharp pain*.' They used phrases like *'sticking large needles into my chest*’ or ‘*stabbing in the chest*’ to articulate their symptoms.

#### Imagine simultaneously scratched by thousands of long nails (p: 33)!

The intensity of the chest symptoms and shortness of breath created a sense of imminent death or a death wish. Participants commonly used phrases, such as ‘*I saw death in front of my eyes’* or ‘*I would rather die than endure the pain*,’ to describe the severity of their pain. Shortness of breath was also a chest symptom, presented in isolation or with other symptoms. Breathlessness mainly occurred at night or during or after physical activity, such as doing house chores.

*For a few days, as soon as I was lying down or doing a chore, I would feel like I was drowning in the sea. That day (the day she came to the hospital), I could barely breathe (p: 11).*

#### Experience of non-chest symptoms

Several women presented with non-chest symptoms. Sweating was the most common non-chest symptom, described as a sudden episode of heavy cold sweating. Pain in the epigastric area, upper back, or wrist, feeling of indigestion, nausea, and vomiting were other symptoms. Some women experienced palpitations. A woman described how she became aware of her heart beating, which trigged her that something was wrong with her heart. Several women developed numbness in different body parts, such as the back, shoulders, neck, or jaw, but the numbness was more common in the left hand. In addition, a limited number of women reported feeling lightheaded, lethargic, dizzy, unusual fatigue, or pale.

*I could not explain how I was feeling. It felt as if I was no longer on the earth, I do not know how to explain it… like dizziness, severe fatigue, lightheadedness, or something like that (p: 39).*

#### Symptomless

In three participants, the ACS condition was detected only by accident; participant No. 19 had had pain in her leg for an extended period and rested because of it. While in the hospital for knee surgery, clinicians detected some abnormal changes in the electrocardiogram and referred her to the emergency department of the participating heart hospital. In addition, two participants were visiting their cardiologists for their hypertension problem when their ACS was detected, and they were referred to the hospital for an emergency angiography.

*My cardiologist called the ambulance himself; he said you should go to the hospital immediately. He spoke with the cardiologist in the hospital himself. I was just confused (p: 14).*

### Response to the symptoms

Participants responded to their symptoms differently, but none of them took their symptoms seriously if the symptoms were not sudden and severe.

#### Using home remedies

Most women attempted to relieve their symptoms by using home remedies (n = 10) if their symptoms were not sudden and severe or they did not think of a cardiac origin. These participants did not consider their symptoms serious enough, which needed immediate medical attention. They assumed that they could treat the symptoms by using some home remedies. For example, women who had attributed their pain to muscular tension used a warm compress or massage as a remedy. They used emollients such as olive oil or blood-boosting oils to ease the pain, like black sesame oil or pepper oil. Women who attributed their symptoms to overeating or having heavy food used yogurt, lemon juice, mint, and horseradish to relieve the symptoms. One participant said:

*I felt like all the food I had eaten was pounding in my esophagus, so I hung from a horizontal bar to let the food down (p: 22).*

Another participant with a similar feeling swallowed large pieces of bread to push down the food *(p: 32).* Women, who attributed their symptoms to low blood sugar levels, tried something sweet, like sugar water or dates.

*I thought my heartburn was because of food…. I was thinking like I shouldn’t have had that meal…I tried mints to remedy the symptoms…. (P: 1)*

#### Ignoring the symptoms

Participants who did not have severe pain or what they considered serious symptoms tended to ignore their symptoms (n = 3). For some women, the pain was part of their life; thus, when ACS occurred, they did not take the symptoms seriously that needed medical care. They expected that the symptoms would resolve if they ignored them.

*I always have pain somewhere… my legs, knees, hands, and now my chest is an overplus (p: 12).*

*In older age, you should not pay too much attention to pain; otherwise, you gotta be in the hospital every day. You always have pain somewhere (p: 27).*

#### Hiding the symptoms

Some participants hid their symptoms from others (n = 4). They expressed various reasons for not disclosing their ACS symptoms. One participant shared that she was ashamed, as her children had to take her to the doctor frequently due to her ill health *(p: 2)*. Similarly, another participant did not want to burden the family (p: 3).

One participant described that she had visited the doctor several times in the past for various reasons, and each time was told that her symptoms did not have a physical origin and were mental health-related. Therefore, the participant was concerned that doctors might have related her symptoms to mental issues again, leading to losing the family’s trust *(p: 36)*.

*I was ashamed to tell them (my sons). They might have said in their hearts that mum is always sick; she is old (she smiles). They wouldn’t say it to be fair, they get along very well, but I do not like to burden (p: 9).*

#### Deciding to seek medical help

A decision to seek medical help was made almost only when women experienced severe symptoms (n = 22). In other words, the main factor that triggered seeking care from health facilities was the severity of the symptoms, especially chest pain.

*I could not even stand my husband putting my clothes on. I was just shouting, hurry up, hurry up (p: 5).*

*“I knew that nothing and no one could help me remedy that deadly pain except the hospital (p: 4).*

Nevertheless, women, who were familiar with the ACS symptoms because they had seen them in a family member before, decided to seek medical care quickly, even if their symptoms were not severe. Two women shared seeking immediate medical help, although their pain was at a moderate level. These women had previously witnessed similar symptoms in their husbands and were aware of the importance of the symptoms and the necessity of early medical interventions.

Although sudden onset, severe pain, and severe symptoms triggered participants to rush to a health center, in cases where the symptoms were mild or bearable, there was a delay from the onset of symptoms to deciding to seek medical help. Seeking medical treatment was delayed until the person could no longer endure the pain or symptoms.

Delay in seeking medical help occurred due to initial hesitation about the necessity of visiting an emergency department; barriers such as living in rural and remote areas also caused a significant delay in accessing timely treatment. As the participating hospital is the primary referral public specialized heart hospital in northwest Iran, some participants had to travel hours to arrive; therefore, they missed the standard gold time for primary interventions. Having negative attitude towards staff in the EDs and patients' reluctance to visit the hospital during the COVID-19 pandemic emerged as other reasons for delaying medical care. These women delayed seeking medical help until their condition became unbearable.

*I said if I go to the hospital, they'll just give me some painkillers; I'll wait to see a specialist after the Corona (p: 20)*

*It was hurting, I knew that something was wrong, but I told myself that I should not go to the hospital in this situation (COVID-19 pandemic)…with my high blood pressure and diabetes, I wouldn’t survive it (if I caught COVID-19) (p: 13*).

### Arriving at the hospital

Participants were brought to the hospital by ambulance or personal vehicle.

#### Arriving by ambulance

Participants brought to the hospital by an ambulance (n = 18) were either referred from a small hospital/medical center or had previous experience using the ambulance service for themselves, a family member, or a relative. Participant No. 6, whose son had passed away only a few days before the interview, had used the ambulance a few times over the preceding days due to her ill health:

*My husband called the ambulance, and they arrived pretty fast. They know our home. During those three days (after her son's death), I frequently felt sick, so we called them several times (p: 6).*

Some patients used the ambulance service because they were familiar with the service. They had called an ambulance for a family member in the past or had heard about using the ambulance for a relative or friend. They knew how the ambulance service operated in Iran and thus felt comfortable calling the ambulance when they experienced ACS symptoms.

*When my son had a (car) accident, bystanders immediately called the ambulance. In the hospital, a nurse told us he would have died of bleeding if you had brought him late. Since then, we have decided to call an ambulance when there is a problem... When I became like that (experienced ACS symptoms), my husband called them immediately (p: 8).*

Some participants used an ambulance because they had regretted not using it in the past and learned from experience to use the ambulance service in medical emergencies.

*When my husband had a stroke, we took her to the hospital ourselves. There, I saw patients brought in by ambulance were receiving quicker care. Since then, we have realized that using an ambulance is very important (p: 10).*

In addition, some participants called an ambulance as they evaluated their condition as ‘critical’, needing immediate medical attention. They believed that the ambulance was a safer option for medical emergencies due to the presence of paramedics and necessary equipment.

*It would be dangerous to come (to the hospital) by ourselves. In an ambulance, you are with a health team member, equipment,….(p: 37)*

#### Using a personal vehicle

Some patients were brought to the hospital by a personal vehicle (n = 21). The participants in this category were two groups; the first group consisted of patients who were unfamiliar with the ambulance service, as they had not used it previously. Therefore, when they experienced ACS symptoms, they did not consider the ambulance an option. Below are excerpts from two participants:

*While I was there, my husband said to my son, help put your mum in the car; it looks like her condition is serious " (p: 19).*

*“We did not think about it (calling the ambulance) at all. Ummu…, they (the family) were so frightened to see me like that (lethargic with frequent vomiting)” (p: 16).*

The second group included patients who held negative attitudes towards the ambulance service. They assumed the ambulance would arrive late and it would be faster if they brought the patient to the hospital by a personal vehicle. Further, some women did not call the ambulance, as they had this wrong assumption that the ambulance service would be costly, while this service is free in Iran. Another reason for not calling an ambulance was the the participants’ uncertainty about their eligibility for the service. Participant 26 said they did not call the ambulance because they were unsure if the ambulance service would evaluate her condition as critical, requiring an ambulance. She recalled when a relative called the ambulance for her Covid-19 condition, but her request was refused for not being critical enough.

*My sister said, "call an ambulance," but my husband said no, by the time the ambulance arrives, we’ll get her to the hospital ourselves (p; 31).*

## Discussion

This study aimed to explore symptom experience and help-seeking behaviors of women who developed ACS for the first time. Participants presented with a diverse range of ACS symptoms, but most women experienced chest symptoms, with or without shortness of breath. Some women, however, presented with non-chest symptoms, such as pain in the epigastric area, upper back, or wrist, sweating, and feeling of indigestion, nausea, and vomiting. Research has shown that experiencing non-chest symptoms is a factor that contributes to delayed help-seeking and access to care and treatment [17].

In the current study, many women attributed their symptoms to a non-cardiac cause, such as acid reflux, fluctuations in blood glucose level, heavy food, distress, COVID-19, lung problems, muscle overuse, and aging. The inaccurate casual attributions resulted in a tendency to underestimate ACS symptoms and take inappropriate actions by women, such as attempting to ease the symptoms with home remedies, which further delayed seeking medical care. This finding also fits with the broader literature suggesting that women tend to deny or underestimate their cardiovascular risk [18]; they are reluctant to identify or admit their risk factors, even those well-known risk factors, such as high blood pressure or high blood cholesterol [19]. In a recent study, less than half of women who had developed acute myocardial infarction or undergone coronary artery bypass grafting attributed their heart condition to their positive family history or smoking behavior [2]. Another factor that could affect treatment seeking behaviors of women in this study is the type of onset of symptoms. Women in our study delayed seeking help if their symptoms were mild and developed gradually. They tended to take these symptoms less seriously. Supporting this finding, O’Donnell et al. reported that patients who experienced slow-onset MI were more likely to attribute their symptoms to a non-cardiac cardiac cause and delay treatment seeking than women who experienced fast-onset MI. This finding has implications for the education of the public about ACS symptoms [20, 21].

Our study included only women who experienced ACS for the first time. This can partially explain the high rate of inaccurate causal attributions made by participants and their delay in seeking medical help. A study in Jordan found that delay in the diagnosis and treatment of ACS was more common in first-time ACS patients than in those with previous ACS experience [22]. When women experience the warning or early symptoms of ACS for the first time, they may focus on symptom relief more than symptom attribution or try to understand the cause of their symptoms through developing various hypotheses. They may try some over- the- counter or home remedies, which result in delayed definitive treatment available at the hospital [18].

In addition, the participant's response to the ACS symptoms was affected by the severity of their symptoms. Women sought immediate medical treatment if they experienced severe and intolerable symptoms regardless of their causal attribution. In contrast, those with mild or moderate symptoms often delayed help-seeking until their symptoms became unbearable. This finding is in line with previous research; prehospital delay is found to be shorter in patients who experience server and abrupt symptoms [15]. Davis also found that pre-hospital delay was more common in women with milder symptoms with a gradual onset [18]. It is vital that the public is educated about the ACS symptoms and encouraged to seek early medical help when they suspect a cardiac cause. Smolderen et al. suggest several potential points in seeking medical care [13]; it is important to identify these points and work on them to promote early help-seeking for ACS.

ACS is a time-sensitive health condition, meaning early treatment can significantly improve disease outcomes. Therefore, using the ambulance service should be encouraged to reduce prehospital delays [15]. In the current study, women who were brought to the hospital by ambulance were referred from another medical facility, had previous experience of using an ambulance for themselves or family members in the past, or had learned from experience to call an ambulance in an emergency. Women, who were unfamiliar with the ambulance service, held misperceptions about this service, such as arriving late or charging for the service, and those who were not sure about their eligibility for using an ambulance, used a personal vehicle to arrive at the hospital. Understanding and addressing factors that affect people's decision to call an ambulance is important in improving ambulance use and reducing prehospital delays for ACS.

## Limitations

This qualitative study recruited participants from a single center in northwest of Iran. Although the participating center was a large tertiary referral hospital, our findings may not be transferable to other women who experience ACS in Iran and other countries. Further, this study recruited only female patients; thus, the results may not be transferable to male patients with ACS.

## Conclusions

This study shed some light on women's experience of the ACS symptoms, their symptom interpretation, and their responses. The findings help identify factors contributing to prehospital delay and affect women's decision-making to seek medical care for ACS. The results support the findings of previous research suggesting that women may experience non-chest symptoms of ACS, attribute their symptoms to a non-cardiac cause, and underestimate their symptoms. These factors delay timely treatment in women. The findings also expand our knowledge of factors contributing to the public's use of the ambulance service for medical emergencies: health professionals and the public need to improve awareness about ACS symptoms in women needs. Women should be supported to develop an accurate symptom attribution and appreciate the importance of early treatment-seeking in the disease outcomes.

## Data Availability

Data will be made available upon request.
